# Single-nucleus transcriptomics reveals a gatekeeper role for FOXP1 in primate cardiac aging

**DOI:** 10.1093/procel/pwac038

**Published:** 2022-09-06

**Authors:** Yiyuan Zhang, Yandong Zheng, Si Wang, Yanling Fan, Yanxia Ye, Yaobin Jing, Zunpeng Liu, Shanshan Yang, Muzhao Xiong, Kuan Yang, Jinghao Hu, Shanshan Che, Qun Chu, Moshi Song, Guang-Hui Liu, Weiqi Zhang, Shuai Ma, Jing Qu

**Affiliations:** National Laboratory of Biomacromolecules, CAS Center for Excellence in Biomacromolecules, Institute of Biophysics, Chinese Academy of Sciences, Beijing 100101, China; State Key Laboratory of Stem Cell and Reproductive Biology, Institute of Zoology, Chinese Academy of Sciences, Beijing 100101, China; State Key Laboratory of Membrane Biology, Institute of Zoology, Chinese Academy of Sciences, Beijing 100101, China; Beijing Institute for Stem Cell and Regenerative Medicine, Beijing 100101, China; State Key Laboratory of Stem Cell and Reproductive Biology, Institute of Zoology, Chinese Academy of Sciences, Beijing 100101, China; University of Chinese Academy of Sciences, Beijing 100049, China; Institute for Stem Cell and Regeneration, Chinese Academy of Sciences, Beijing 100101, China; Beijing Institute for Stem Cell and Regenerative Medicine, Beijing 100101, China; Aging Translational Medicine Center, Xuanwu Hospital, Capital Medical University, Beijing 100053, China; Advanced Innovation Center for Human Brain Protection, National Clinical Research Center for Geriatric Disorders, Xuanwu Hospital Capital Medical University, Beijing 100053, China; The Fifth People’s Hospital of Chongqing, Chongqing 400062, China; CAS Key Laboratory of Genomic and Precision Medicine, Beijing Institute of Genomics, Chinese Academy of Sciences, Beijing 100101, China; China National Center for Bioinformation, Beijing 100101, China; State Key Laboratory of Stem Cell and Reproductive Biology, Institute of Zoology, Chinese Academy of Sciences, Beijing 100101, China; Institute for Stem Cell and Regeneration, Chinese Academy of Sciences, Beijing 100101, China; Beijing Institute for Stem Cell and Regenerative Medicine, Beijing 100101, China; State Key Laboratory of Membrane Biology, Institute of Zoology, Chinese Academy of Sciences, Beijing 100101, China; University of Chinese Academy of Sciences, Beijing 100049, China; Institute for Stem Cell and Regeneration, Chinese Academy of Sciences, Beijing 100101, China; Beijing Institute for Stem Cell and Regenerative Medicine, Beijing 100101, China; State Key Laboratory of Stem Cell and Reproductive Biology, Institute of Zoology, Chinese Academy of Sciences, Beijing 100101, China; University of Chinese Academy of Sciences, Beijing 100049, China; Institute for Stem Cell and Regeneration, Chinese Academy of Sciences, Beijing 100101, China; Beijing Institute for Stem Cell and Regenerative Medicine, Beijing 100101, China; Aging Translational Medicine Center, Xuanwu Hospital, Capital Medical University, Beijing 100053, China; Advanced Innovation Center for Human Brain Protection, National Clinical Research Center for Geriatric Disorders, Xuanwu Hospital Capital Medical University, Beijing 100053, China; University of Chinese Academy of Sciences, Beijing 100049, China; CAS Key Laboratory of Genomic and Precision Medicine, Beijing Institute of Genomics, Chinese Academy of Sciences, Beijing 100101, China; China National Center for Bioinformation, Beijing 100101, China; University of Chinese Academy of Sciences, Beijing 100049, China; CAS Key Laboratory of Genomic and Precision Medicine, Beijing Institute of Genomics, Chinese Academy of Sciences, Beijing 100101, China; China National Center for Bioinformation, Beijing 100101, China; Sino-Danish College, University of Chinese Academy of Sciences, Beijing 101408, China; Aging Translational Medicine Center, Xuanwu Hospital, Capital Medical University, Beijing 100053, China; Advanced Innovation Center for Human Brain Protection, National Clinical Research Center for Geriatric Disorders, Xuanwu Hospital Capital Medical University, Beijing 100053, China; University of Chinese Academy of Sciences, Beijing 100049, China; CAS Key Laboratory of Genomic and Precision Medicine, Beijing Institute of Genomics, Chinese Academy of Sciences, Beijing 100101, China; China National Center for Bioinformation, Beijing 100101, China; State Key Laboratory of Stem Cell and Reproductive Biology, Institute of Zoology, Chinese Academy of Sciences, Beijing 100101, China; Institute for Stem Cell and Regeneration, Chinese Academy of Sciences, Beijing 100101, China; Beijing Institute for Stem Cell and Regenerative Medicine, Beijing 100101, China; The Fifth People’s Hospital of Chongqing, Chongqing 400062, China; State Key Laboratory of Membrane Biology, Institute of Zoology, Chinese Academy of Sciences, Beijing 100101, China; University of Chinese Academy of Sciences, Beijing 100049, China; Institute for Stem Cell and Regeneration, Chinese Academy of Sciences, Beijing 100101, China; Beijing Institute for Stem Cell and Regenerative Medicine, Beijing 100101, China; National Laboratory of Biomacromolecules, CAS Center for Excellence in Biomacromolecules, Institute of Biophysics, Chinese Academy of Sciences, Beijing 100101, China; State Key Laboratory of Membrane Biology, Institute of Zoology, Chinese Academy of Sciences, Beijing 100101, China; University of Chinese Academy of Sciences, Beijing 100049, China; Institute for Stem Cell and Regeneration, Chinese Academy of Sciences, Beijing 100101, China; Beijing Institute for Stem Cell and Regenerative Medicine, Beijing 100101, China; Advanced Innovation Center for Human Brain Protection, National Clinical Research Center for Geriatric Disorders, Xuanwu Hospital Capital Medical University, Beijing 100053, China; University of Chinese Academy of Sciences, Beijing 100049, China; Institute for Stem Cell and Regeneration, Chinese Academy of Sciences, Beijing 100101, China; Beijing Institute for Stem Cell and Regenerative Medicine, Beijing 100101, China; CAS Key Laboratory of Genomic and Precision Medicine, Beijing Institute of Genomics, Chinese Academy of Sciences, Beijing 100101, China; China National Center for Bioinformation, Beijing 100101, China; Advanced Innovation Center for Human Brain Protection, National Clinical Research Center for Geriatric Disorders, Xuanwu Hospital Capital Medical University, Beijing 100053, China; Sino-Danish College, University of Chinese Academy of Sciences, Beijing 101408, China; State Key Laboratory of Membrane Biology, Institute of Zoology, Chinese Academy of Sciences, Beijing 100101, China; University of Chinese Academy of Sciences, Beijing 100049, China; Institute for Stem Cell and Regeneration, Chinese Academy of Sciences, Beijing 100101, China; Beijing Institute for Stem Cell and Regenerative Medicine, Beijing 100101, China; The Fifth People’s Hospital of Chongqing, Chongqing 400062, China; State Key Laboratory of Stem Cell and Reproductive Biology, Institute of Zoology, Chinese Academy of Sciences, Beijing 100101, China; University of Chinese Academy of Sciences, Beijing 100049, China; Institute for Stem Cell and Regeneration, Chinese Academy of Sciences, Beijing 100101, China; Beijing Institute for Stem Cell and Regenerative Medicine, Beijing 100101, China

**Keywords:** single-nucleus RNA-sequencing, primate, aging, FOXP1, cardiomyocyte

## Abstract

Aging poses a major risk factor for cardiovascular diseases, the leading cause of death in the aged population. However, the cell type-specific changes underlying cardiac aging are far from being clear. Here, we performed single-nucleus RNA-sequencing analysis of left ventricles from young and aged cynomolgus monkeys to define cell composition changes and transcriptomic alterations across different cell types associated with age. We found that aged cardiomyocytes underwent a dramatic loss in cell numbers and profound fluctuations in transcriptional profiles. Via transcription regulatory network analysis, we identified FOXP1, a core transcription factor in organ development, as a key downregulated factor in aged cardiomyocytes, concomitant with the dysregulation of FOXP1 target genes associated with heart function and cardiac diseases. Consistently, the deficiency of FOXP1 led to hypertrophic and senescent phenotypes in human embryonic stem cell-derived cardiomyocytes. Altogether, our findings depict the cellular and molecular landscape of ventricular aging at the single-cell resolution, and identify drivers for primate cardiac aging and potential targets for intervention against cardiac aging and associated diseases.

## Introduction

Aging is widely known as a premier risk factor for cardiovascular diseases (CVD), including coronary heart disease, hypertension, ischemic disease, and heart failure (Stern et al., 2003; Marian and Braunwald, 2017). As aging, the heart tissue undergoes hypertrophic growth, an adaptive state that exacerbates cardiac pathophysiology and leads to compensatory mechanisms that increases vulnerability to stress, thereby reducing ability to maintain an efficient circulation and heightening risk of heart failure (Li et al., 2020). Of the four chambers of the heart, the left ventricle (LV), located in the bottom left portion, is responsible for pumping oxygenated blood into the aorta, and from then on to all tissues in the body. As LV compartment is critical to the health of the entire body, and particularly susceptible to aging (Lakatta, 2003; Lakatta and Levy, 2003), uncovering regulatory mechanisms that control LV aging is crucial to deepen our understanding of cardiac aging and unravel novel intervention strategies.

The LV is a complex chamber composed of multiple cell types, primarily cardiomyocytes, but also fibroblasts, endothelial cells, cardiac conduction system cells (such as neuronal cells and Purkinje cells), and immune cells (Litvinukova et al., 2020; Tucker et al., 2020; Wang et al., 2020a; Leng and Pawelec, 2022). All of these cell types undergo differential phenotypic, cellular and molecular changes in response to aging (Obas and Vasan, 2018). The LV becomes thick and enlarged due to an increase in cardiomyocyte size, despite the number of cardiomyocytes lost due to cell death or senescence (Abdellatif et al., 2018; Li et al., 2020). Aged cardiomyocytes also exhibit diverse epigenetic and transcriptional changes, and imbalanced protein homeostasis (Shimizu and Minamino, 2016; Cui et al., 2018; Triposkiadis et al., 2019; Zhao and Chen, 2022). A series of molecular mechanisms that exacerbate cardiomyocyte aging have been reported, such as activation of the TGF-β signaling pathway or the Renin–Angiotensin–Aldosterone system, a decreased response to β-adrenergic signaling, and dysregulation of the PI3K/AKT/mTOR pathway (Cowan and Young, 2009; Wang et al., 2010; Lyu et al., 2018; Hu et al., 2020; Marin-Aguilar et al., 2020). In addition, immune cells, such as T and B cells, are intimately involved in aging-associated inflammation and tissue remodeling in heart (Ramos et al., 2017; Dick et al., 2019; Abplanalp et al., 2021; Leng and Pawelec, 2022). In this context, gene expression profiling of the aged LV at single-cell resolution holds significant promise toward dissecting its cellular diversity and aging-associated mechanisms.

Given ethical restrictions, it is very difficult to obtain human LV tissues from aged-matched and healthy individuals. Non-human primates (NHPs), such as cynomolgus monkeys, are genetically very close to humans. Consequently, their hearts are structurally and functionally similar to those in humans, and even display aging-related cardiovascular disease risk markers conserved in humans (Greiter-Wilke et al., 2016). Since LV tissues from healthy and aged monkey can be sampled and comprehensively investigated, NHPs serve as an ideal model for cardiac aging research.

Herein, we established the first single-nucleus transcriptomic atlas for LV tissues from both young and aged healthy monkeys. Based on the datasets, we identified cell type-specific differentially expressed genes, as well as gene regulatory networks associated with NHP LV aging. Furthermore, we revealed and validated the role of FOXP1, a master regulator in antagonizing cardiac aging in monkey LV tissues and human cardiomyocytes. Overall, our study provides a comprehensive understanding of primate LV aging at a single-cell resolution, thereby facilitating development of new therapeutic strategies targeting cardiac aging and age-associated cardiovascular diseases.

## Results

### Age-related physiological and morphological features in cynomolgus monkey LV

**Figure 1.**
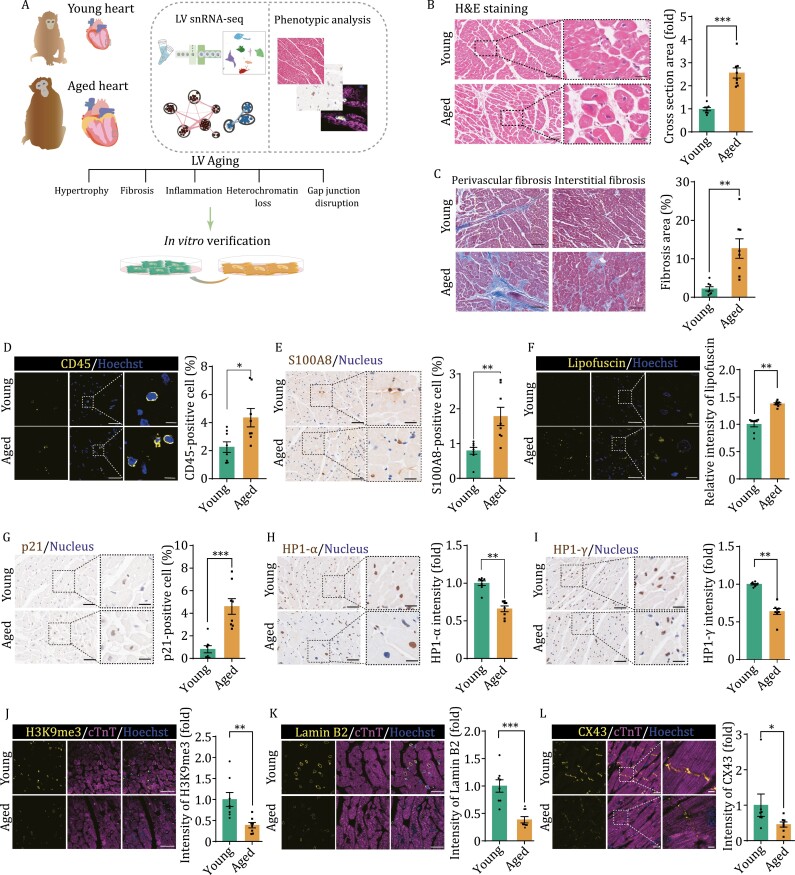
Characterization of aging-associated physiological changes in the monkey left ventricle. (A) Schematic flowchart overview of samples, analysis, and validation approaches. Young, young cynomolgus monkeys, 4–6 years old; Aged, old cynomolgus monkeys, 18–21 years old. (B) Hematoxylin and eosin (H&E) staining of LV tissues from young and aged monkeys. Scale bars, 100 μm and 25 μm (zoomed-in images). Left, representative images. Right, quantitative analysis. (C) Masson staining of the LV tissues from young and aged monkeys. Scale bars, 100 μm. Left, representative images for perivascular fibrosis and interstitial fibrosis as indicated. Right, quantitative analysis. (D) Immunofluorescence staining for CD45 in LV tissues from young and aged monkeys. Scale bars, 50 μm and 10 μm (zoomed-in images). Left, representative images. Right, the proportion of CD45 positive cells was quantified. (E) Immunohistochemical staining for S100A8 in LV tissues from young and aged monkeys. The proportion of S100A8-positive cells in total cells was calculated as quantitative analysis. Scale bars, 50 μm and 20 μm (zoomed-in images). Left, representative images; right, the proportion of S100A8 positive cells was quantified. (F) Analysis for lipofuscin deposition in LV tissues from young and aged monkeys. Scale bars, 50 μm and 10 μm (zoomed-in images). Left, representative images; right, the relative intensity was quantified as fold changes of their intensity in the aged LV vs. that in young LV tissues. (G) Immunohistochemical staining for cellular senescence-associated marker p21 in LV tissues from young and aged monkeys. Scale bars, 50 μm and 20 μm (zoomed-in images). Left, representative images. Right, the proportion of p21 positive cells was quantified. (H) Immunohistochemical staining for heterochromatin protein HP1-α in LV tissues from young and aged monkeys. Scale bars, 50 μm and 20 μm (zoomed-in images). Left, representative images. Right, the relative intensity was quantified as fold changes of their intensity in the aged tissues vs. that in young tissues. (I) Immunohistochemical staining of heterochromatin protein HP1-γ in LV tissues from young and aged monkeys. Scale bars, 50 μm and 20 μm (zoomed-in images). Left, representative images. Right, the relative intensity was quantified as fold changes of their intensity in the aged tissue vs. that in young tissue. (J) Immunofluorescence staining of cTnT and H3K9me3 in LV tissues from young and aged monkeys. Scale bars, 50 μm. Left, representative images. Right, the relative intensity was quantified as fold changes of their intensity in the aged samples vs. that in young samples. (K) Immunofluorescence staining of cTnT and Lamin B2 in LV tissues from young and aged monkeys. Scale bars, 25 μm. Left, representative images. Right, the relative intensity was quantified as fold changes of their intensity in the aged samples vs. that in young samples. (L) Immunofluorescence staining of cTnT and CX43 in LV tissues from young and aged monkeys. Scale bars, 25 μm and 5 μm (zoomed-in images). Left, representative images. Right, the relative intensity was quantified as fold changes of their intensity in the aged samples vs. that in young samples. Data are presented as the mean ± SEM. *n* = 8 monkeys for each group. *P < 0.05, **P < 0.01; ***P < 0.001.

To assess physiological alterations of the LV associated with cardiac aging, we obtained LV tissues from 8 young (4–6 years old) and 8 aged (18–21 years old) cynomolgus monkeys, which are equivalent to young (20 years old) and aged (60 years old) humans (Fig. 1A). Although the weight ratio of the heart and the whole body was slightly increased, there was no significant change in heart rate (Fig. S1A) (Zhang et al., 2020). In H&E-stained sections, we observed a 2.5-fold increase in the cardiomyocyte cross-sectional area in aged animals relative to control, a classic indicator of hypertrophic and aged cardiomyocytes (Fig. 1B). In Masson’s trichrome stained sections, we found that the fibrotic areas at both interstitial and peri-vascular sites of aged LV tissues were augmented (Fig. 1C). These data suggest that the LV tissue in aged monkey undergoes remodeling, consistent with development of cardiac fibrosis and hypertrophy with age. Notably, we observed elevated immune cell infiltration in aged LV tissues, as evidenced by a larger percentage of CD45-positive immune cells relative to young LV tissues (Fig. 1D). Consistently, S100A8, which is normally expressed in activated macrophages or neutrophils in response to inflammatory stimuli (Wang et al., 2018; Cai et al., 2020), was elevated in aged LV (Fig. 1E). We also detected the deposition of cellular lipofuscin granules, another typical sign of aging (Kakimoto et al., 2019), in the aged tissues (Fig. 1F). Moreover, we observed other senescence-associated changes in aged LV tissues, including increased p21-positive cells (Fig. 1G), and downregulation of heterochromatin-associated proteins (HP1-α and HP1-γ, Fig. 1H and 1I) and heterochromatin-associated histone mark H3K9me3 (Fig. 1J), as well as nuclear lamina protein Lamin B2 (Fig. 1K). In addition, CX43, a gap-junction protein important for intercellular signaling in cardiac muscle, was also downregulated in the aged LV tissues (Fig. 1L). Altogether, these results indicate that the aged primate LV tissues exhibit senescent phenotypes and progressive architecture decay.

### Single-nucleus transcriptomic profiling of NHP LV aging

**Figure 2.**
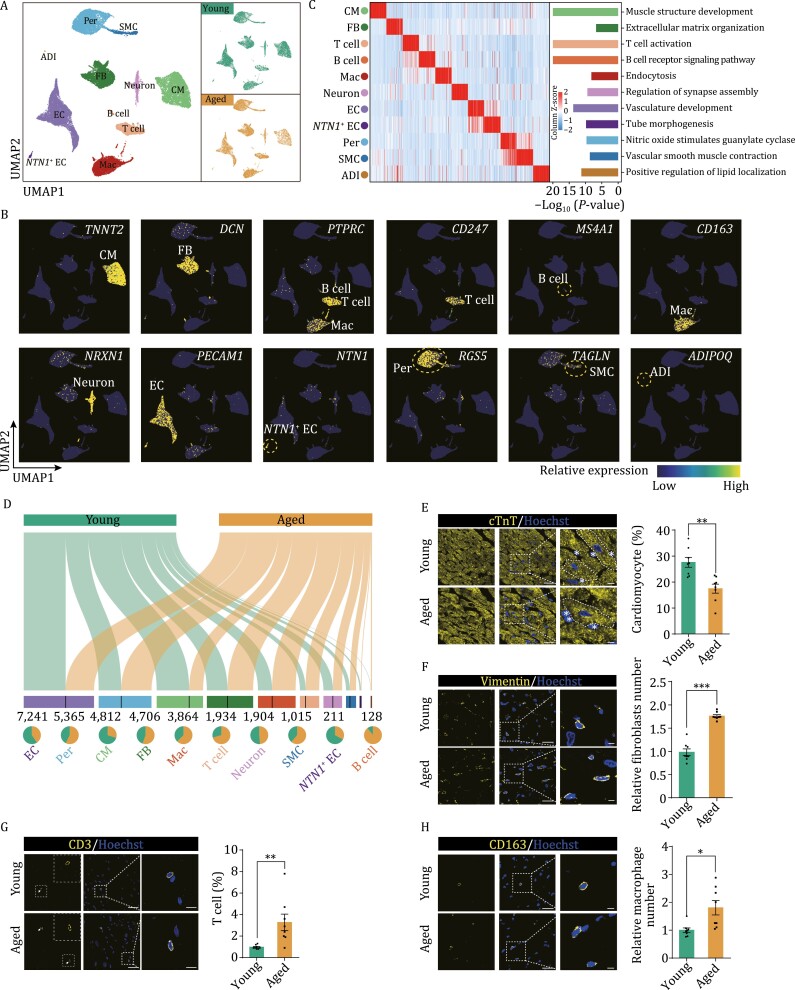
Single-cell atlas of left ventricle revealed the changes of cell proportion in the aged heart. (A) UMAP (uniform manifold approximation and projection) plot showing the distribution of different cell types in the LV tissues of young and aged monkeys. CM, cardiomyocyte; FB, fibroblast; T cell, T lymphocyte; B cell, B lymphocyte; Mac, macrophage; Neuron; EC, endothelial cell; *NTN1*^+^ EC, *NTN1*-postivite endothelial cell; Per, pericyte; SMC, smooth muscle cell; ADI, adipocyte. (B) UMAP plots showing the expression profiles of indicated cell-type-specific marker genes in monkey heart. The color key from blue to yellow indicates low to high gene expression levels. (C) Left, heatmap showing the expression signatures of top 50 cell-type-specific genes. Enriched representative GO terms and pathways for each cell type are showing on the right. Colors indicate different cell types and the length of bar indicates −log_10_ (*P*-value). (D) Sankey plot showing the number of cells and the ratios of young and aged cells in each cell type. The length of the bar indicates the number of cells, and the number of cells was marked below the bar. The pie chart showing the ratios of cell types in young and aged monkey LV. (E) Immunofluorescence staining of cardiomyocyte marker cTnT in young and aged monkey LV. Scale bars, 25 μm and 5 μm (zoomed-in images). Left, representative images. Right, the percentage of cardiac nuclei to total nuclei was calculated, the asterisk in representative images indicates non-cardiomyocytes. (F) Immunofluorescence staining of fibroblast marker Vimentin in young and aged monkey LV. Scale bars, 25 μm and 5 μm (zoomed-in images). Left, representative images. Right, the percentage of vimentin positive cells to total nuclei was quantified. (G) Immunofluorescence staining of T cell marker CD3 in young and aged monkey LV. Scale bars, 50 μm and 10 μm (zoomed-in images). Left, representative images. Right, the percentage of CD3 positive cells to total nuclei was quantified. (H) Immunofluorescence staining of macrophage marker CD163 in young and aged monkey LV. Scale bars, 25 μm and 5 μm (zoomed-in images). Left, representative images. Right, the percentage of CD163 positive cells to total nuclei was quantified. Data are presented as the mean ± SEM. *n* = 8 monkeys for each group. *P < 0.05, **P < 0.01, ***P < 0.001.

To comprehensively dissect the cellular responses to LV aging, we obtained 35,612 nuclei from snap-frozen LV samples collected from 3 young and 3 aged male cynomolgus monkeys for single-nucleus RNA-sequencing (snRNA-seq). We assigned 31,205 high-quality nuclei after stringent quality control into 11 cell types with distinct cellular transcriptomic signatures upon principal component analysis (PCA) dimension reduction followed by graph-based clustering, and visualization by uniform manifold approximation and projection (UMAP) (Figs. 2A and S1B–F; Table S1). Enrichment analysis of marker genes across diverse cell types revealed features corresponding to known biological functions and characteristics of each cell cluster (Figs. 2B, 2C and S2A). For example, CM marker genes are associated with muscle structure development (Fig. 2C). Through this approach, we successfully identified cardiomyocytes (CM), fibroblasts (FB), T cells, B cells, macrophages (Mac), pericytes (Per), endothelial cells (EC), and smooth muscle cells (SMC). Of note, we also identified cell types that are typically not recovered from single-cell dissociation by enzyme digestion, such as neuron and adipocytes (ADI) (Fig. 2A; Table S1).

Next, we analyzed the dynamics of aging-associated cardiac cell composition and found that the proportions of cardiomyocytes and EC trended towards a decrease, while the proportions of fibroblasts, SMC and immune cells increased with age (Figs. 2D and S2B). In order to verify the changes in cell compositions in the aged LV tissues, we performed immunofluorescence staining with cell-specific markers. Indeed, we found that the proportion of cTnT-positive cardiomyocytes dropped about one third in aged LV tissue when comparing with young LV (Fig. 2E). By contrast, the relative number of fibroblasts in aged LV was about 1.7 times higher than that in young individuals, consistent with the augmented fibrosis we had observed (Figs. 1C and 2F). Moreover, immune cell infiltration, including T cells and macrophages, was increased in aged LV (Fig. 2G and 2H). Collectively, these observations suggest a loss of functional cell types and a shift towards fibroblast accumulation and immune cell infiltration in the aged LV, likely contributing to hypertrophic, fibrotic, and inflammatory alterations in the aged NHP LV.

### Cell type-specific alterations in transcriptional regulatory programs of aged NHP LV

**Figure 3.**
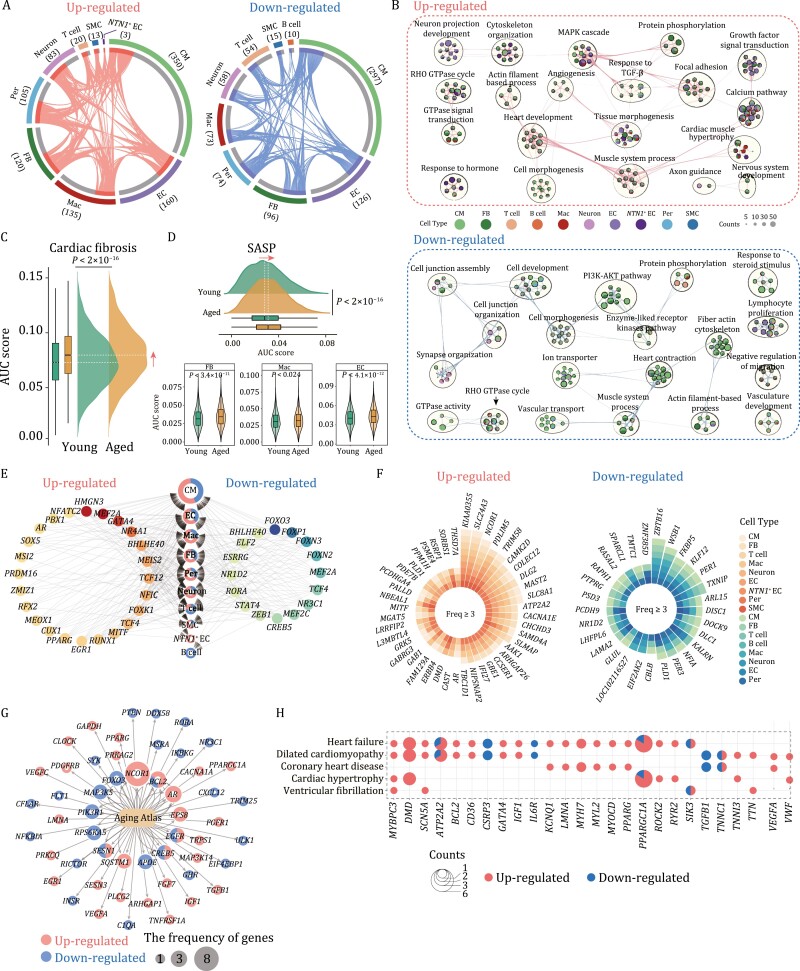
Transcriptional changes in multiple cell types during cardiac aging. (A) Circos plots showing up (left) and downregulated (right) aging-related differentially expressed genes (aging DEGs) in different cell types in monkey LV. Each connecting line represents a DEG that co-occurs in both cell types. (B) Network visualizing representative GO terms and pathways of aging-related up (top) and downregulated (bottom) DEGs between aged and young monkey LV cells. The nodes were represented as pie charts, where the size of a pie is proportional to the total number of hits that fall into that specific term. The pie charts are colored by cell types, where the size of a slice represents the percentage of genes under the term that originated from the corresponding gene list. Two terms with similarity > 0.3 are connected by a line. (C) Ridge plot showing the AUC score of cardiac fibrosis gene set in fibroblasts. (D) Ridge plot showing the AUC score of senescence-associated secretory phenotype (SASP) gene set in the monkey LV cells. Violin plots showing increased SASP genome scores in FB, Mac, and EC in monkey LV during aging. (E) Network visualization of aging-related up (left) and downregulated (right) core regulatory transcription factors (TFs) in monkey ventricular cells. The pie charts in the middle represent different cell types, with red representing upregulated and blue representing downregulated. The node size indicates the number of target genes involved in a certain cell type. The red nodes on the left represent upregulated TFs, and the blue nodes on the right represent downregulated TFs. Color keys from light to dark indicate the numbers of target genes regulated by these TFs from low to high. (F) Plots showing up (left) and downregulated DEGs (right) shared by at least three cell types. The color key indicates different cell types. (G) Network plot showing up and downregulated DEGs of all cell types that overlapped with genes annotated in the Aging Atlas database. The node size indicates the frequency of DEGs appearing across different cell types. Red parts of nodes, upregulated genes; blue parts of nodes, downregulated genes. (H) Dot plots showing that DEGs overlapped with genes from heart disease-associated gene sets. Red parts of nodes, upregulated DEGs; blue parts of nodes, downregulated genes. The node size indicates the frequency of DEGs.

To discriminate between common and unique gene expression changes associated with aging in each LV cell type, we characterized aging-related differentially expressed genes (DEGs) across the major cell types in young and aged samples. We identified 1,792 aging-related DEGs (|log_2_FoldChange| > 0.25 and adjusted *P* value < 0.05) that were differentially expressed in at least one cell type in young and aged LV (Fig. 3A). Major cell types in heart, included CM, EC, and FB, were most affected by aging, as manifested by high DEG counts of 647, 286 and 216 respectively (Fig. 3A). Although we observed a high degree of variability in aging DEGs across different cell types, some GO term annotations were shared across the major cell types (Fig. 3B; Table S2). For example, the downregulated DEGs were enriched in cell junction organization (*ANK3, TJP1*, *JUP*), synapse organization (*ANK3, NRXN1, NLGN1, NEGR1*), heart contraction (*ATP2A, RYR2, MYL2*), and the PI3K-AKT pathway (*ANGPT1, EIF4EBP1, FOXO3, INSR*), consistent with age-associated functional decline (Fig. 3B; Table S2). Upregulated DEGs were associated with pro-fibrotic signaling, such as TGF-β signaling-associated genes (*TGFB1, LTBP1, ANKRD1*) and other cardiomyopathy-related signaling, such as the MAPK cascade (*ADRA1A, EGR1, IL6R*) (Figs. 3B and S2C; Table S2). Consistently, the gene set score (AUCell) of fibroblasts demonstrated an activation of genes related to cardiac fibrosis (Fig. 3C; Table S3). Furthermore, a focused inspection also revealed augmented expression of genes related to the senescence-associated secretory phenotype (SASP), a classic characteristic of aged tissues (Fig. 3D; Table S3). These results underscore the development of both pro-fibrotic and pro-inflammatory transcriptional signatures in the aged LV.

Next, we asked whether disturbed cellular communications between CM and other cell types in LV samples were present in the aged heart. When we applied to bioinformatic tool iTALK (Wang et al., 2019) to predict cell–cell communication, we detected various changes in cellular interactions of the aged versus young CM (Fig. S2D and S2E). Overall, cell–cell communications were decreased when assessing interaction between CM, pericyte, SMC and other cell types, which suggests structural disorganization (Fig. S2D). This drop in interactions was partially caused by a profound aging-associated reduction in the *TIE1* receptor (Fig. S2E), that when mutated causes malfunction of Angiopoietin/Tie2 signaling and severe cardiovascular defects (Venkatesh et al., 2008). In sharp contrast, the interactions between ligands of ECs and receptors of other cell types (for example, fibroblasts) were enhanced in aged LV (Fig. S2D). Aged ECs are predicted to express factors associated with endothelial injury and inflammation, such as *VCAM1, ICAM4, MMP9, SPP1* and *ADAM12* (Fig. S2E), which potentially mediates abnormal cell–cell interactions between EC and various cell types in the aged LV. Taken together, these results reveal the pro-inflammatory and pro-fibrotic state of the aged LV, consistent with the histopathological results (Fig. 1C and 1D).

### Core transcription factor and hotspot genes underlying primate LV aging

To untangle transcription mediators regulating LV aging, we performed SCENIC analysis to predict core transcription factors (TFs) across different cell types of the aged LV (Figs. 3E and S3A). These core TFs include *MITF* (TF for aging DEGs in macrophage, fibroblast, and pericyte), a gene linking to the hypertrophic response (Mehta et al., 2015), and *AR* (TF for aging DEGs in CM, macrophage, and fibroblast), a key molecular target to treat heart failure (Fig. 3E). We also identified that downregulation of *NR1D2*, a gene involved in congenital heart disease (Priest et al., 2016), was a feature of aging-related transcriptional network in aged macrophage, fibroblast and pericyte (Figs. 3E and S3A). Interestingly, co-downregulated TFs across multiple cell types also revealed that multiple members of the forkhead transcription factor family were present in aged cells (*FOXO3, FOXP1, FOXN3, FOXN2*) (Figs. 3E and S3A).

We also identified high-frequency aging DEGs that were shared in at least three cell types (Fig. 3F). For example, *SLC24A3*, a previously reported high blood pressure-related gene (Citterio et al., 2011; Georges et al., 2021), was increased in nine cell types (e.g., CM, fibroblast, and EC). *CAMK2D,* and *CACNA1E,* that are associated with abnormal calcium handling and arrhythmias respectively, were increased in multiple types of cardiac cells (e.g., CM, neuron, and fibroblast). *ZBTB16*, for which expression is highly correlated with mitochondrial numbers, was decreased in seven cell types (e.g., CM, fibroblast, and T cell). The downregulated DEGs were also involved in the circadian rhythm (*PER1, PER3, NR1D2*) (Fig. 3F).

Next, in joint analysis of DEGs and the Aging Atlas database (Aging Atlas, 2021), we pinpointed *SQSTM1* (also known as p62) (aging DEG in CM and fibroblast), a proteotoxic stress sensor, and *BCL2* (aging DEG in CM, fibroblast, and EC), an apoptosis mediator, as genes that were increased in aged LV (Fig. 3G; Table S3). *TGFB1* (aging DEG in EC), which is involved in the formation of myocardial fibrosis and promotes cardiac hypertrophy, as upregulated gene in the aged LV (Fig. 3G; Table S3). Conversely, mTOR signaling genes *EIF4EBP1* and *RICTOR* were decreased in the aged heart which may underlie coincident compromised cardiac physiology and metabolism (Fig. 3G; Table S3). Within the databases comprising hotspot genes involved in cardiovascular diseases, we identified a set of DEGs that overlapped with those genes related to cardiac hypertrophy, coronary heart disease, heart failure, dilated cardiomyopathy, and ventricular fibrillation, resolving the pro-disease state of the aged LV at the transcription level (Figs. 3H and S3B; Table S3). Collectively, these data provide insights into the transcription regulatory network for cardiac LV aging, and pinpoint key player for LV degeneration.

### FOXP1 was identified as a core transcriptional regulator for CM aging

**Figure 4.**
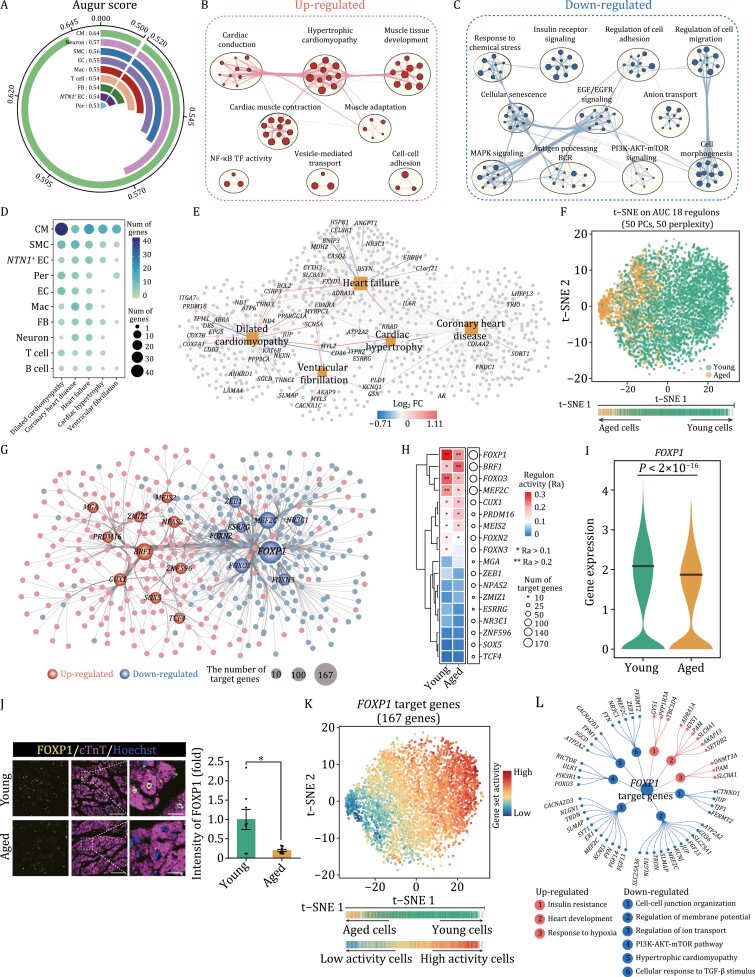
Profiling of aging-susceptible cardiomyocyte changes. (A) Circle plot showing prioritization of the most responsive cell types during monkey heart aging by Augur (a method to identify the cell types most responsive to biological perturbations in single-cell data). (B) Network visualizing representative GO terms and pathways of upregulated aging-related DEGs between aged and young monkey cardiomyocytes. The size of nodes is proportional to the total number of hits that fall into a certain specific term. Two terms with similarity > 0.3 are connected by a line. (C) Network visualizing representative GO terms and pathways of downregulated aging-associated DEGs between aged and young monkey cardiomyocytes. The size of nodes is proportional to the total number of hits that fall into a certain term. Two terms with similarity > 0.3 are connected by a line. (D) Dot plot showing the number of genes that overlap between marker genes of each cell types and cardiovascular disease-associated gene sets. (E) Network visualizing the overlap between aging DEGs of cardiomyocytes and genes involved in cardiovascular diseases. The colors of grey and yellow nodes represent genes and diseases, respectively. Among nodes of genes, DEGs of cardiomyocytes are labeled by gene symbols, with edges indicating log_2_ fold changes. Red, upregulation; blue, downregulation. (F) t-SNE plot showing cluster distribution of monkey cardiomyocytes by the 18 aging-related regulons activity. Cells are colored by young and old groups. (G) Regulatory networks visualizing potential key transcriptional regulators in monkey cardiomyocytes during aging. Smaller nodes represent target genes and larger nodes represent TFs. The node size of TFs positively correlates with the number of target genes it regulates. Red nodes, upregulated; blue nodes, downregulated. (H) Plot showing regulon activity and number of target genes of 18 aging-related regulators in CMs. The size of the dots was positively correlated with the number of target genes. (I) Violin plot showing the expression of *FOXP1* in monkey cardiomyocytes from young and aged groups, indicated that *FOXP1* is downregulated in aged cardiomyocytes. The black line represents the median expression. (J) Immunofluorescence staining of FOXP1 in young and aged monkey LV, verifying the downregulation of FOXP1 in aged cardiomyocytes, but not other cell types. Left, representative image of FOXP1 in aged and young heart. Right, quantitative data of FOXP1 expression in cardiomyocytes. Scale bars, 25 μm and 5 μm (zoomed-in images). Data are presented as the mean ± SEM. *n* = 8 monkeys for each group. **P < 0.01. (K) t-SNE plot showing activity score of the *FOXP1* target gene set in monkey cardiomyocytes. (L) Network plot showing representative terms of *FOXP1* target gene enrichment. The genes corresponding to the terms are shown on the outermost side. Red nodes, upregulated; blue nodes, downregulated.

To deepen our insight into the transcriptomic landscape of LV aging, we next performed Augur analysis (Skinnider et al., 2021), a method to identify the cell types most responsive to biological perturbations in single-cell data, and found that CM was the cell type most affected by aging (Fig. 4A). As the changes in cell ratio and transcription profiles also suggested that CM plays a major role in cardiac LV aging, and CM conducts primary contractile function of the heart, we focused on exploring biological implication of CM aging DEGs. To this end, we conducted gene enrichment analysis, and observed stronger enrichments of upregulated genes involved in hypertrophic cardiomyopathy (*ADRA1A, TNNI3, MYL2*) and cardiac muscle contraction (*CACNA1C, CASQ2, CAMK2B*) (Park et al., 2018; Ng et al., 2020) (Fig. 4B; Table S2). In contrast, downregulated genes were enriched in growth-related EGF/EGFR signaling (*BRAF, MEF2C, EIF4EBP1*) (Tang et al., 2017; Hashimoto et al., 2019; Waliany et al., 2021) and cell morphogenesis (*FOXO3, EIF4EBP1, PI3KR1*) (Ruan et al., 2021) (Fig. 4C; Table S2), indicating a structural and functional deterioration of CM during aging. Consistently, we found that hotspot genes associated with age-related cardiac diseases (coronary heart disease, dilated cardiomyopathy, heart failure and ventricular fibrillation, etc.) were highly enriched in CM relative to other cell types (Fig. 4D; Table S3). We also observed strong associations between aging-related genes expression and the cardiac diseases-associated genes in aged CM (Figs. 4E and S3C; Tables S2 and S3). These results suggest that aging-associated changes in gene expression in CM closely correlate with susceptibility to cardiovascular diseases in the elderly.

In transcription factor (TF) regulatory network analysis, we identified 18 young and aged CM-specific regulons whose expression levels distinguished young and aged cells (Fig. 4F). Among them, the FOXP1 regulon was downregulated in aged primate CM, ranking as a top transcription factor controlling aging-DEGs of primate CM (Figs. 4G, 4H and S3D). We also found that *FOXP1* transcription level was reduced in the aged LV and verified downregulation of FOXP1 in aged CM by immunofluorescence (Fig. 4I, and 4J). According to sequencing data, FOXP1 acts broadly upstream of 167 aging-associated DEGs in CM, and consequently, these target genes were disturbed in aged CM along with decreased expression of FOXP1 (Fig. 4K). Specifically, upregulated FOXP1 target genes were associated with heart development (*ADRA1A, AKAP13, SLC8A1*), insulin resistance (*GYS1, PPP1R3A, TBC1D4*) (Xirouchaki et al., 2016), and response to hypoxia (*DNMT3A, PAM, SLC8A1*); downregulated FOXP1 target genes were related to cell–cell junction organization (*JUP, TJP1*), regulation of ion transport (*FGF13, KCNJ3, CACNA2D3*) and regulation of membrane potential (*ATP2A2, KCNJ3, CD36*) (Fig. 4L). These data suggest that repression of FOXP1 and disturbances of its downstream transcriptional network likely contribute to the cardiomyocyte aging in monkey LV.

### Knockdown of *FOXP1* induced pro-hypertrophic remodeling in human cardiomyocytes

**Figure 5.**
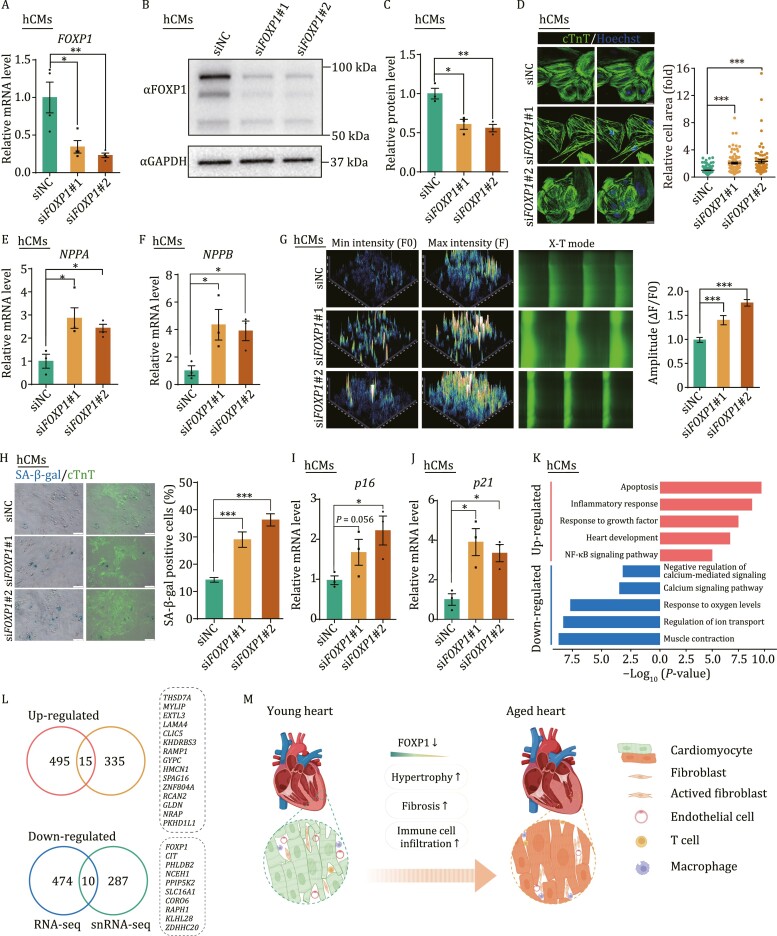
Downregulated FOXP1 induced senescence and hypertrophy in hCMs. (A) RT-qPCR detecting silent efficacy of FOXP1 siRNA in hCMs. Data are presented as mean ± SEM. *n* = 4 independent experiment, *P < 0.05, **P < 0.01. (B) Western blot detecting FOXP1 protein levels in hCMs after transfection of siNC and si*FOXP1*#1 and si*FOXP1*#2. (C) Quantitative data of (B) showed the downregulation of FOXP1 protein level after transfected with si*FOXP1*#1 and si*FOXP1*#2 compared with siNC. Data are presented as mean ± SEM. *n* = 3 independent experiment, *P < 0.05, **P < 0.01. (D) Immunofluorescence staining of cTnT in hCMs after transfection of siNC and si*FOXP1*#1 and si*FOXP1*#2. Left, representative photos. Right, quantitative data. Scale bars, 20 μm. Data are presented as mean ± SEM. *n* = 3 independent experiment, ***P < 0.001. (E) RT-qPCR detecting the cardiac hypertrophic marker *NPPA* after transfection of siNC and si*FOXP1*#1 and si*FOXP1*#2. Data are presented as mean ± SEM. *n* = 3 independent experiment, *P < 0.05, **P < 0.01. (F) RT-qPCR detecting the cardiac hypertrophic marker *NPPB* after transfection of siNC and si*FOXP1*#1 and si*FOXP1*#2. Data are presented as mean ± SEM. *n* = 3 independent experiment, *P < 0.05. (G) Calcium transient recordings and quantification of amplitude by Fluo-4 AM in hCMs upon knockdown of FOXP1. Left, representative photos showing the *F*0 (min intensity) and *F* (max intensity) of calcium transients in hCMs transfected with siNC, si*FOXP1*#1 and si*FOXP1*#2. Middle: X-T mode of each group. Right: quantitative data. Data are presented as mean ± SEM. *n* = 3 independent experiment, **P < 0.01. (H) Co-staining of SA-β-gal activity and cTnT immunofluorescence in hCMs after transfection of siNC and si*FOXP1*#1 and si*FOXP1*#2. Left, representative photos. Right, quantitative data. Data are presented as mean ± SEM. *n* = 3 independent experiment, ***P < 0.001. (I) RT-qPCR detecting the senescence marker *p16* after transfection of siNC and si*FOXP1*#1 and si*FOXP1*#2. Data are presented as mean ± SEM. *n* = 3 independent experiment, *P < 0.05. (J) RT-qPCR detecting the senescence marker *p21* after transfection of siNC and si*FOXP1*#1 and si*FOXP1*#2. Data are presented as mean ± SEM. *n* = 3 independent experiment, **P < 0.01. (K) Representative GO terms and pathways of up- and downregulated DEGs in siNC and si*FOXP1* hCMs by RNA-sequencing. (L) Venn diagram showing genes shared by DEGs of hCMs after silencing of FOXP1 and aging-related DEGs of cardiomyocytes in snRNA-seq. (M) Schematic (created with Biorender.com) showing pathological changes in the LV of young and aged hearts.

To investigate the functional role of *FOXP1* in cardiomyocytes, we knocked down FOXP1 with siRNAs in human cardiomyocytes (hCMs) differentiated from human embryonic stem cells (hESCs). After siRNA transfection, we validated FOXP1 reduction at both RNA and protein expression levels by RT-qPCR and western blot (Fig. 5A–C). *FOXP1-*knockdown hCMs were larger than control hCMs, a phenotype accompanied by increased expression of cardiac hypertrophy markers *NPPA* and *NPPB* (Fig. 5D–F). Notably, hCMs with FOXP1 knockdown displayed calcium overload, and had a retarded calcium transient, suggesting accumulation of cardiac damage (Fig. 5G). More importantly, knockdown of *FOXP1* in hCM simultaneously resulted in accelerated senescence, as characterized by increased SA-β-gal activity and increased expression of the senescence markers *p16* and *p21* (Fig. 5H–J). At the transcriptomic level, our RNA-seq data demonstrate that FOXP1 silencing induced a transcriptomic signature resembling the one we had observed in aged CMs with snRNA-seq (Fig. S3E and S3F; Table S4). For example, upregulated genes were associated with apoptosis, inflammation, and the NF-κB signaling pathway, whereas downregulated genes were related to muscle contraction, ion transport, and calcium signaling (Fig. 5K; Table S4). Notably, a few DEGs overlapped between *FOXP1*-knockdown hCM and aged monkey CM, including 15 upregulated genes and 10 downregulated genes that are closely related to physiological homeostasis of CM (Fig. 5L; Tables S2 and S4). Altogether, these results provide a molecular fingerprint of how deficiency of *FOXP1* result in the pro-hypertrophic remodeling of hCMs, mirroring the aging phenotypes observed in LV tissues from aged monkeys.

## Discussion

In this study, we used single-nucleus RNA sequencing to delineate the transcriptional landscape of cardiac LV tissue from young and aged cynomolgus monkeys. We determined the composition dynamics of different cell populations and characterized the differential gene expression profiles associated with aging for each cell type. We found that the most pronounced aging-related changes occurred in CM, and that FOXP1 was the primary regulatory mediator of DEGs in aged CM, given that FOXP1 was downregulated in aged monkey CM and hCMs acquired hypertrophic and senescence phenotypes upon FOXP1 silencing. This work complements existing transcriptomic landscapes and expands our knowledge on the specific changes that occur during cardiac aging.

Massively single-cell/nucleus sequencing are powerful technologies that have been successfully applied to generate detailed knowledge of cellular signatures and functional states in fresh or frozen tissues (Ma et al., 2021; Zhang et al., 2021). For complex tissues, such as the adult mammalian heart, snRNA-seq is preferred as it overcomes the technical challenge of isolating intact single cells from complex tissues, a requirement for scRNA-seq (Wang et al., 2020a, 2020c; Yuan et al., 2021). The other advantage of single-nucleus sequencing is that it spans all cell types present in complex organs, such as cardiomyocytes, fibroblasts, ECs, macrophages, SMCs and so on, as reported in this study and previous related studies (Hu et al., 2018; Cui et al., 2020; Tucker et al., 2020). For example, an earlier study that analyzed adult human cardiac tissue specimens sampled from six anatomical heart regions, described the heterogeneity of cardiomyocytes with diverse developmental origins and specialized properties (Litvinukova et al., 2020). Our study extends from earlier work in that we establish a robust single-nucleus transcriptome resource that allows us to compare cellular states of all major cardiac cell types between the young and aged primate LV. Together with previous studies (Sun et al., 2022; Wang et al., 2022a, 2022b), our work will help improve our understanding of the biology of the human heart and will provide a valuable resource for future studies.

FOXP1 is a transcriptional repressor that binds numerous promotors and enhancers through its forkhead DNA-binding domain. During heart development, FOXP1 regulates myocyte proliferation and maturation, as well as cardiac cushion development upstream of SOX4 (Zhang et al., 2010). As such, FOXP1 is expressed in both the myocardium and the endocardium and regulates transcription across cardiomyocytes, ECs, and vascular SMCs (Zhang et al., 2010; Bot et al., 2011; Grundmann et al., 2013). In the absence of FOXP1, cardiomyocyte proliferation is increased and differentiation is decreased, leading to increased cardiac mass, and ultimately to neonatal death (Zhang et al., 2010). Here, we found, for the first time, that the deficiency of FOXP1 drives human CM aging, revealing an unappreciated geroprotective role of FOXP1 in primate heart aging. Interestingly, the dysregulation of FOXP1 has been reported to be associated with cardiovascular diseases, including cardiac hypertrophy, coronary heart disease, heart failure and dilated cardiomyopathy (Bai and Kerppola, 2011; Yang et al., 2015; Liu et al., 2019, 2022; Zhuang et al., 2019). For instance, FOXP1 inhibits the expression of genes related to hypertrophy such as ANP, BNP, and MYH7 (Bai and Kerppola, 2011). The downregulation of FOXP1 by miR-206/YAP also leads to the development of cardiac hypertrophy (Yang et al., 2015). Moreover, knockdown of FOXP1 in EC has been reported to induce fibrosis and myocardial remodeling via the upregulation of the expression of TGF-β, and ultimately lead to heart failure (Liu et al., 2019). Recently, FOXP1 has also been reported to associate with aging. For instance, in bone marrow mesenchymal stem cells, FOXP1 expression decreases with age and is inversely correlated with p16 expression, and reduced FOXP1 expression in aged mesenchymal stem cells is a driver of skeletal aging (Li et al., 2017). Additionally, FOXP1 has been found to be inactivated in the aged human ovary (Cicek et al., 2013).

Mechanistically, our results indicate that FOXP1 counteracts apoptosis and inflammation in human CM. Consistent with these findings, FOXP1 was reported to regulate vessel inflammation by directly regulating endothelial inflammasome components, including Nlrp3, caspase-1, and IL-1β (Zhuang et al., 2019). Similarly, endothelial-specific Foxp1 overexpression in mice reduces atherosclerotic lesion formation and monocyte infiltration (Li et al., 2021). Altogether, our data and earlier work suggest that targeting FOXP1 could be a mechanism that delays aging in multiple tissues, particularly in cardiovascular tissues. It should be noted that there are several limitations in the present study. First, how FOXP1 is downregulated in aged monkey cardiomyocytes, especially its regulation at the epigenetic level, requires further investigation. The answer to this question may help us develop new intervenable targets or pathways to delay LV aging. Secondly, the specific downstream target genes of FOXP1 and the underlying molecular mechanisms, by which FOXP1 regulates calcium overload, cardiomyocyte hypertrophy, and cellular senescence remain unclear, and awaits further investigation. Thirdly, further comparative studies on LV in aged monkeys and humans will consolidate species-conserved key molecular mediators underlying cardiac aging, and expand the clinical significance of this study.

In summary, based on our single-cell atlas of non-human primate LV and human cardiomyocyte aging research platform, we identify FOXP1 as a novel gatekeeper of aging in the primate heart. Our work provides an in-depth understanding of cardiac aging, and serves as a comprehensive resource for informing the development of novel therapeutic interventions to treat aging-associated cardiovascular diseases.

## Methods

### Ethical statement

The cynomolgus monkeys used in this study have been approved by the Ethics Review Committee of the Institute of Zoology of the Chinese Academy of Sciences (Zhang et al., 2020).

### Tissue acquisition and sample preparation

Cynomolgus monkeys in the anesthetized state were subjected to phosphate-buffered perfusion. Afterwards, the heart was collected, and then the LV was divided from the heart tissue according to the tissue anatomy.

### Hematoxylin and eosin staining (H&E staining)

The H&E staining was performed following a previous study (Zou et al., 2021). The paraffin-embedded tissue blocks were sectioned, and each tissue section was 5 μm thick. The sections were placed in xylene for 10 min each time, three times in total to remove the paraffin. After dewaxing, the tissue sections were immersed in graded alcohol rehydration (100%, 100%, 90%, 70%, 50%). Subsequent immersion in distilled water for 5 min twice. The slices were then stained with hematoxylin for 5 min and rinsed by running water for 2 min. The hematoxylin staining was differentiated with ethanol containing 1% hydrochloric acid and rinsed by running water for 2 min. The tissue was then stained with eosin solution for 3 min and washed by distilled water, and then rapidly dehydrated and rendered transparent in xylene. Photos were taken by PerkinElmer Vectro Polaris, and the cross-section area of cardiomyocytes was quantified using Image Pro plus.

### Masson’s trichrome staining

Masson’s trichrome staining was performed using the Masson Trichrome Staining Kit (Solarbio, G1340) following the protocol as previously described (Lei et al., 2021). Paraffin sections were dewaxed and a hematoxylin staining solution was used to stain the nuclei. After nuclei staining, the sections were stained with a combination of ponceau 2R, acidic fuchsin and glacial acetic acid staining solution. Then the samples were treated with 1% phosphomolybdic acid solution, then re-stained with 2% aniline blue solution. 1% glacial acetic acid solution in water for 1 min, then dehydrated twice in 95% alcohol, twice in anhydrous ethanol, rendered transparent in xylene. Photos were taken by PerkinElmer Vectro Polaris, and the fibrosis area, including peri-vascular fibrosis area and interstitial fibrosis area, was quantified using Image Pro plus.

### Tissue immunohistochemistry and immunofluorescence

Immunofluorescence and immunohistochemistry experiments were performed using paraffin sections with a section thickness of 5 μm as previously described (Wang et al., 2020b). After dewaxing and rehydrating the paraffin sections, the heat antigen retrieval step was performed with a citrate-based antigen retrieval solution (PH 6.0). Then permeabilized with PBS containing 0.4% Triton X-100 for 30 min at RT. After washing with PBS buffer, endogenous peroxide was blocked with hydrogen peroxide blocker (ZSGB-Bio) for immunohistochemistry only (immunofluorescence staining was not done at this step). The samples were then blocked with 10% donkey serum. Overnight incubation with primary antibody (antibody list was shown in Table S5) was performed at 4°C. For immunofluorescence staining, secondary antibody incubation together with Hoechst staining was performed the next day at RT for 1 h. Leica TCS SP8 confocal microscope was used for immunofluorescence photography. For immunohistochemistry, a secondary antibody (ZSGB-Bio) was used to develop DAB chromogenic solution, followed by hematoxylin staining of nuclei. Photos were taken by PerkinElmer Vectro Polaris, and statistics were quantified using Image Pro plus and Image J.

### Generation of human cardiomyocyte from hESCs

Differentiation of hESCs into human Cardiomyocytes (hCMs) were performed as previously described based on a S12 supplements dependent approach (Gu et al., 2014; Lee et al., 2017; Wang et al., 2022a; Zhang et al., 2022). The S12 medium was an albumin-free and chemical-defined supplement for cardiac differentiation including fatty acid, protein, chemicals, and antioxidants (Zhao et al., 2017). In brief, hESCs were dissociated into small clusters by 0.5 mmol/L EDTA and cultured onto Matrigel-coated plate in mTeSR medium. The hCM differentiation was initiated once hESCs reaching to about 90% confluency. For hCM induction, the culture medium was switched to RPMI 1640 basal medium supplemented with S12 (without insulin) and 5 μmol/L CHIR99021 on day 0. After 24 h, the medium was replaced by RPMI 1640 basal medium supplemented with S12 (without insulin) only. Then, 5 μmol/L IWR-1 was added since day 3. The differentiation medium was then switched to RPMI 1640 basal medium containing S12 with insulin from day 5. The cardiomyocytes subsequently appeared to beat from day 7 to 9.

### RNA quantification

Cultured cells and LV tissues stocked in liquid nitrogen were taken for RNA extraction using TRIzol Reagent (Wu et al., 2020; Chu et al., 2022). LV tissues were ground with a tissue grinder, and then TRIzol (Invitrogen) was added, mixed thoroughly at RT. The other steps were then performed following the previous report (Wu et al., 2020). The reverse transcription was carried out using reverse transcription kit (Vazyme, R323-01). The qPCR reactions were carried out in a 5 μL reaction volume containing 2.5 μL SYBR^®^ Green Realtime PCR Master Mix (Toyobo, QPK-201), 0.2 μL of cDNA, 0.2 μL of forward and reverse primers, respectively (Primer list was shown in Table S6). Reactions were performed in ABI QuantStudio 5 (Applied Biosystems, Thermo-Fisher Scientific). Only samples with CT values < 40 in two or more replicates were considered positive. Samples with indeterminate CT values or with CT > 40 in at least two replicates were considered negative.

### Western blot

The western blot analysis was performed as previously described (Ma et al., 2022). Briefly, cell pellets or the ground LV tissue were lysed in RIPA lysate (Beyotime, P0013B) supplemented with protease inhibitors (Roche, 4693159001). After being full lysed, the samples were centrifuged and the supernatant of the lysate was aspirated and quantified with the BCA kit (Dingguo biotechnology, BCA02). SDS-PAGE electrophoresis was followed by semi-dry membrane transfer. After being transferred, the PVDF membranes (Merck Millpore) were blocked with 5% skim milk for 1.5 h and then incubated with primary antibody overnight at 4°C. The following day, after being washed by TBST buffer, the PVDF membrane was incubated with the secondary antibodies conjugated with HRP according to the source of the primary antibody and incubated at RT for 1 h. The image was obtained with ChemiDoc XRS+ system (Bio-Rad Laboratories). The band intensity was quantified using Image J.

### Cell immunofluorescence

The protocol of cell immunofluorescence was followed the previous study (Liang et al., 2022). In brief, cells seeded on Matrigel-coated coverslips were washed with PBS to remove residual culture medium and then fixed in 4% PFA for 10 min at RT. Cells were then washed twice in PBS buffer and permeabilized with PBS containing 0.4% Triton X-100 for 5 min at RT. Cells were washed twice in PBS and blocked in 10% donkey serum for 30 min at RT. Incubation with primary antibodies were performed overnight at 4°C. The next day, the samples were washed and incubated with fluorescent-dye conjugated secondary antibodies at RT for 1 h. The images were photographed with Zeiss LSM880.

### siRNA transfection

The cardiomyocytes were digested into single cells and cultured in 12-well plates or six-well plates for more than 24 h, and then transfected with siRNAs against human *FOXP1* (si*FOXP1*#1, si*FOXP1*#2) or negative control (siNC) (Rebobio, Table S6) using Lipofectamine™ 3000 Transfection Reagent (Invitrogen) following the manufacturer’s instructions.

### SA-β-gal and immunofluorescence co-staining

For SA-β-Gal staining, hCMs were fixed in 2% formaldehyde and 0.2% glutaraldehyde at RT for 5 min and stained with freshly prepared staining solution at 37°C for 9–10 h (X-gal was purchased from Amresco, all the other reagents were from Sigma-Aldrich) (Bi et al., 2020). After SA-β-gal staining, cells were permeabilized with PBS containing 0.4% Triton X-100 for 5 min at RT. Subsequently, cells were blocked with 10% donkey serum for 30 min. The cardiomyocytes were incubated with primary antibody (cTnT, Abcam) overnight at 4°C, and then the fluorescent-dye conjugated secondary antibody at RT for 1 h. Images were taken with an Olympus CKX41 microscope, and the percentages of SA-β-gal-positive cells were quantified using Image J.

### Calcium transient and calcium sparks

hESC derived hCMs were transfected with siNC and si*FOXP1*#1/si*FOXP1*#2 for 96 h followed by calcium transient assay as previous study described (Ahola et al., 2018). The hCMs were incubated with 2.5 μmol/L Fluo-4 AM for 30 min, followed by two washes by HBSS. Ca^2+^ transient was monitored from a central region of the cell (avoiding the nucleus) in line scan (X-T) mode using Zeiss LSM880 with excitation at 488 nm and emission recorded from 500 to 590 nm. For the amplitude quantification of calcium transient, we first captured the images using time series mode, and then quantified the maximum fluorescence intensity value (*F*) and minimal fluorescence intensity value (*F*0). The amplitude was calculated by the formula as follow: Amplitude = (*F*−*F*0)/*F*0.

### snRNA-seq library preparation

Cardiac nuclei isolation followed the previously published approach (Krishnaswami et al., 2016; Zhang et al.*,.* 2021). In brief, the frozen LV tissues were ground into homogenates and centrifuged to obtain nuclei. After Hoechst and PI staining, the nuclei were sorted by flow cytometry. All snRNA-seq libraries were prepared using Chromium Single-Cell 3ʹ v3 Reagent Kit (10× Genomics) according to the manufacturer’s protocol. Indexed libraries were equimolarly pooled and sequenced on Illumina NovaSeq 6000 using paired-end 150 bp as sequencing mode by GenomeScan (Leiden, Netherlands).

### Preprocessing of snRNA-seq raw data

Cell Ranger Single-Cell Software Suite (version 3.1.0, 10× Genomics) was used for sample de-multiplexing, barcode processing and single-cell gene counting for snRNA-seq raw data. Briefly, fastq files were aligned to the *Macaca fascicularis* pre-mRNA reference genome (Macaca_fascicularis_5.0, Ensembl) with cellranger counts. A digital gene expression matrix was generated by STAR aligners. To remove the background RNA bias, we used CellBender (version 0.2.0) (Fleming et al., 2019), a tool for background noise removal based on an unsupervised deep generative model.

### snRNA-seq data analysis and cell type identification

The R package Seurat (version 4.1.0) (Hao et al., 2021) was used for downstream analysis of single-nucleus sequencing data, including quality control, dimensionality reduction, cell clustering, and differential expression gene analysis. Specifically, quality control metrics included between 300 and 4,000 genes per cell, and no more than 5% mitochondrial genes. To eliminate the effect of a technical artifact known as “doublets”, the DoubletFinder (version 2.0.3) (McGinnis et al., 2019) software was used to identify and remove double cells in each sample. According to the doublet ratio of the 10x Genomics single-cell platform “resulting in the recovery of ~1000 cells with a fold rate of ~0.8%”, the doublet formation rate was set to “doubletate = cellnum × 8 × 10^−6^”. Finally, 31 205 high-quality nuclei were used for downstream bioinformatics analysis. In order to better eliminate the false positive of biological heterogeneity caused by technical factors such as limitation of sequencing depth by single-nucleus RNA-sequencing approach, we used SCTransform (version 0.3.2) (Hafemeister and Satija, 2019) to normalize and scale the expression matrix. The “PrepSCTIntegration” and “FindIntegrationAnchors” functions were used to select integration anchors and perform downstream integration. These anchors were then used to integrate the dataset of all samples with “IntegrateData” function. The “RunPCA” function was used for principal component analysis, and the top 30 principal components were used for subsequent analysis. “RunUMAP” function was used to perform dimensionality reduction and visualization. The “FindNeighbors” and “FindClusters” functions were used to perform cell clustering. The marker genes for each cluster were determined with the Wilcoxon rank-sum test by “FindAllMarkers” function. Only those with “|avg_log_2_FC|” > 0.5 and “p_val_adj” < 0.05 were considered marker genes (Table S1). Cell types were identified based on expression of classical marker genes.

### Determination of the purity of the cell type

The entropy-based statistic, ROGUE (Ratio of Global Unshifted Entropy) (Liu et al., 2020), was used to accurately quantify the purity of identified cell types in monkey ventricular single-nucleus RNA-sequencing data.

### Identification of aging-related differentially expressed gene

To identify aging-related DEGs (aging DEGs) between young and old groups for each cell type of the monkey ventricle, we used the “FindMarkers” function in Seurat. Cell types with fewer than five cells in each group, such as adipocytes (ADI), were filtered out prior to differential expression analysis. Only genes with adjusted *P*-value < 0.05 (non-parametric Wilcoxon rank-sum test) and |avg_log_2_FC| > 0.25 were considered to be aging-related DEGs (Table S2).

### Pathway enrichment analysis and visualization

A complete protocol (Reimand et al., 2019) and Metascape (Zhou et al., 2019) were used to perform pathway enrichment analysis and visualization of DEGs. The protocol firstly used g:Profiler to perform pathway enrichment analysis on a list of DEGs. Next, the pathway enrichment results were visualized by using EnrichmentMap (version 3.3.1) in Cytoscape (version 3.7.2). Finally, AutoAnnotate (version 1.3.2) in Cytoscape was used to navigate and interpret pathway enrichment results. Metascape is an online portal that provides a comprehensive resource for gene list annotation and analysis.

### Identification of cell types affected by perturbations of aging

Augur (Skinnider et al., 2021), a versatile method to prioritize cell types on the basis of their molecular response to a biological perturbation, was used to identify cell types affected by perturbations of aging. Briefly, we used the “calculate_auc” function in Augur, taking a normalized matrix as input by Seurat and a data frame containing metadata for cell type and age annotations.

### Transcriptional regulatory network analysis

To analyze transcriptional regulatory networks during LV aging, the R package SCENIC (single-cell regulatory network inference and clustering) (version 1.1.2.2) workflow and the cisTarget database were used to predict regulators, which are transcription factors (TFs) and their co-expressed motifs and significantly enriched target genes. Briefly, we first construct TF-genes co-expression networks through GENIE3 (gene network inference with ensemble of trees) (version 1.6.0), a method to infer gene regulatory networks based on the gene expression matrix. Secondly, we download the gene-motif ranking and motif-TF annotation database related to human hg19 from the cisTarget database, and then use RcisTarget (version 1.4.1) software to select significantly enriched motifs and predict its target genes, then TF-gene modules and target gene prediction results are integrated to construct gene regulatory network modules (regulons) of TF and target genes. Finally, the R package AUCell (version 1.8.0) was used to sort all the genes in the above regulators in each cell according to their expression levels from high to low, and calculate the cumulative area under the curve (AUC) based on the gene ranking to evaluate the activity score of the above regulators in each cell. Finally, the TF module networks were visualized by Cytoscape (version 3.7.2).

### Gene set score analysis

R package AUCell (version 1.8.0) was used to score pathway activity in individual cells. Based on the normalized expression matrix by Seurat, the “AUCell_buildRankings” function with default parameters was used to rank the gene expression for each cell. The “AUCell_calcAUC” function was used to calculate the area under the curve (AUC) values based on the gene expression ranking. Heart disease-related gene sets and myocardial fibrosis gene sets were downloaded from the DisGeNET database (Table S3).

### Cell-cell communication analysis

To identify cell–cell communication in ventricular cells during aging, we used the R package iTALK (Wang et al., 2019), an algorithm for identifying and illustrating underlying cell–cell signaling communication networks in single-cell sequencing data. iTALK can identify significant changes, i.e., gains or losses in interactions between young and aged groups by finding and ranking differentially expressed ligands and/or receptors. We used the receptor-ligand database of iTALK, which contains a total of 2,648 non-redundant and known ligand-receptor pairs. Based on the main functions of the ligands, the ligand-receptor database is annotated into four modules: cytokine/chemokine-receptor pairs, growth factor-receptor pairs, immune checkpoint-receptor pairs, and other ligand-receptor pairs. For this analysis, we followed the official workflow to load the expression matrix normalized by the Seurat workflow and meta data into iTALK and build an iTALK object. Differentially expressed genes between young and old groups in each cell type were identified by “DEG” function (method = “Wilcox”, |logFC| ≥ 0.25 and *q*.value < 0.05). The “FindLR” function was used to identify potential differentially expressed ligand-receptor pairs in young and old groups. The chord diagram shows the top 100 ligand-receptor pairs.

### RNA-seq library construction and sequencing

Total RNA was extracted from 1 × 10^5^ cells using Trizol according to the manufacturer’s instructions. The quality and quantity of total RNA were assessed by Fragment Analyzer (AATI) and NanoDrop ND-1000 (Wilmington), respectively. The mRNA was isolated from 2 μg of total RNA using the NEBNext Poly (A) mRNA Magnetic Isolation Module. Subsequently, isolated mRNA was used for RNA library construction using NEBNext Ultra RNA library prep kit for Illumina. The generated libraries were pooled and sequenced on Illumina HiSeq X ten platform with paired-end 150-bp sequencing.

### RNA-seq data analysis

Trim Galore (version 0.4.5) software was used for automate adapter trimming and quality control, and Hisat2 (version 2.0.4) (Kim et al., 2015) with default parameters was used to map the cleaned reads to the UCSC hg19 human genome. HTSeq (version 0.6.1) (Anders et al., 2015) software was used to count the number of reads mapped in each annotated gene based on the mapping results. R package DESeq2 (version 1.2.4) (Love et al., 2014) was used to calculate DEGs with the cutoff values of Benjamini-Hochberg adjusted *P* value (*P*.adjust) < 0.05 and |Log_2_ (fold change)| > 0.58 (Table S4).

### Statistics

The difference between young and aged group were performed using student’s *t*-test. All statistical analyses were performed by Prism 8 software (GraphPad). Data were expressed as mean ± SEM. Differences were considered significant when *P* < 0.05. *P < 0.05, **P < 0.01, ***P < 0.001.

## Supplementary Material

pwac038_suppl_Supplementary_Materials

pwac038_suppl_Supplementary_Table_S1

pwac038_suppl_Supplementary_Table_S2

pwac038_suppl_Supplementary_Table_S3

pwac038_suppl_Supplementary_Table_S4

pwac038_suppl_Supplementary_Table_S5

pwac038_suppl_Supplementary_Table_S6

## Abbreviations

ADI: adipocyte
B cell: B lymphocyte
CM: cardiomyocyte
CVD: cardiovascular diseases
DEGs: differentially expressed genes
EC: endothelial cell
FB: fibroblast
FOXP1: forkhead Box P1
LV: left ventricle
Mac: macrophage
NHP: non-human primate
PCA: principal component analysis
Per: pericyte
SASP: senescence-associated secretory phenotype
SMC: smooth muscle cell
TF: transcription factor
T cell: T lymphocyte
t-SNE: t-distributed stochastic neighbor embedding
UMAP: uniform manifold approximation and projection
