# Supplementary material for: Single-nucleus transcriptomics reveals a gatekeeper role for FOXP1 in primate cardiac aging
Source: Protein Cell. 2022 Sep 6;14(4):279–93. doi: 10.1093/procel/pwac038 (PMC10120965; doi:10.1093/procel/pwac038)
Supplement: pwac038_suppl_Supplementary_Materials [file pwac038_suppl_supplementary_materials.pdf]

Supplemental Materials

Figure S1. Quality control of snRNA-seq data

A. The ratio of heart weight to body weight and the ratio of heart weight to lung weight were measured in young and aged cynomolgus monkeys. Data are presented as the mean ± SEM. n = 8 monkeys for each group.
B. The distribution of quality controls, including median number of genes, sequencing saturation, and mapping rate to the genome, in the snRNA-seq data of each sample.
C. Scatter plot showing the proportion of mitochondrial genes for each cell. The color key from gray to red indicates less to more percentage of mitochondrial genes. Low quality cells are colored by gray.
D. Histograms showing the number of genes detected in individual cells.
E. Histograms showing the number of unique molecular identifiers (UMIs) detected in individual cells.
F. Boxplot showing the value of ROGUE (A method for accurately quantify the purity of an identified cell clusters) of each cell type. Box shows the median and the quartile range (25-75%) and the length of whiskers represents 1.5× the IQR.

Figure S2. DEG analysis in multiple cell types and cell-cell communication

A. Dot plots showing the expression of representative genes for each cell type in monkey LV.
B. Bar plot showing changes in the proportion of cells between young and old monkey LV.
C. Bar plots showing aging-related upregulated (left) and downregulated (right) DEG enriched GO terms or pathways in the seven cell types with the highest DEG numbers in the monkey LV.
D. Network plots showing changes in the number of ligand-receptor interactions detected between each two cell types and/or within a single cell type in the old vs. young groups. The changes in the number of ligand-receptor interactions are indicated by connection lines. The thickness of the lines is positively correlated with the number of ligand-receptor interactions. Red lines indicated an increase in the number of ligand-receptor interactions in the old group, and blue lines indicated a decrease.
E. The circle plot showing the top 100 highly expressed ligand-receptor interactions in the old vs. young groups. The gene types of ligands and receptor are colored by yellow, green and black, and are annotated on the right. The ligand-receptor interaction events are indicated by connecting lines with arrows. The thickness of the line is positively correlated with the expression level of the ligand, and the size of the arrow is positively correlated with the expression level of the receptor. Red lines indicated upregulated ligand and/or receptor in the old group, blue lines indicated ligand-receptor pairs downregulated in the old group.

Figure S3. **Single-nucleus transcriptomic signatures in NHP CM and bulk RNA-seq quality control in primate CM**

A. The heatmap showing the expression levels of aging-related core regulatory transcription factors (TFs) in monkey ventricular cells, with red representing up-regulated and blue representing down-regulated. The size of the dot plot indicates the number of target genes regulated by a certain transcription factor.
B. Dot plots showing that DEGs overlapped with genes from specific heart disease-associated gene sets. Red parts of nodes, upregulated genes; blue parts of nodes,

downregulated genes. The node size indicates the frequency of DEGs.

C. Dot plot showing cardiomyocytes aging-related DEGs overlapped with genes from the heart disease-related gene sets. Red nodes, upregulated genes; blue nodes, downregulated genes.
D. Heatmap showing the activity (row z-score) of 18 aging-related regulators in monkey cardiomyocytes. Top panel, age of each cardiomyocyte (green, young group; yellow, old group).
E. Heatmap showing the relative expression level (row-scaling) of DEGs in siNC and siFOXP1 hCMs by RNA-sequencing.
F. Volcano plot showing the DEGs of siNC and si*FOXP1* hCMs by bulk RNA-sequencing.

Supplemental Table Legends

Table S1. Marker genes for each cell type in cynomolgus monkey left ventricle.

Table S2. Aging-related differentially expressed genes (DEGs) for each cell type in the cynomolgus monkey left ventricle.

Table S3. Gene list for the genes annotated in the Aging Atlas database, cardiac fibrosis-related genes, SASP related genes, and heart disease-related gene sets.

Table S4. Differentially expressed genes (DEGs) in hCMs upon silence of FOXP1.

Table S5. The list of antibodies used in this study.

Table S6. Primer list for qRT-PCR and sequence of siFOXP1#1, siFOXP1#2.

Figure S1

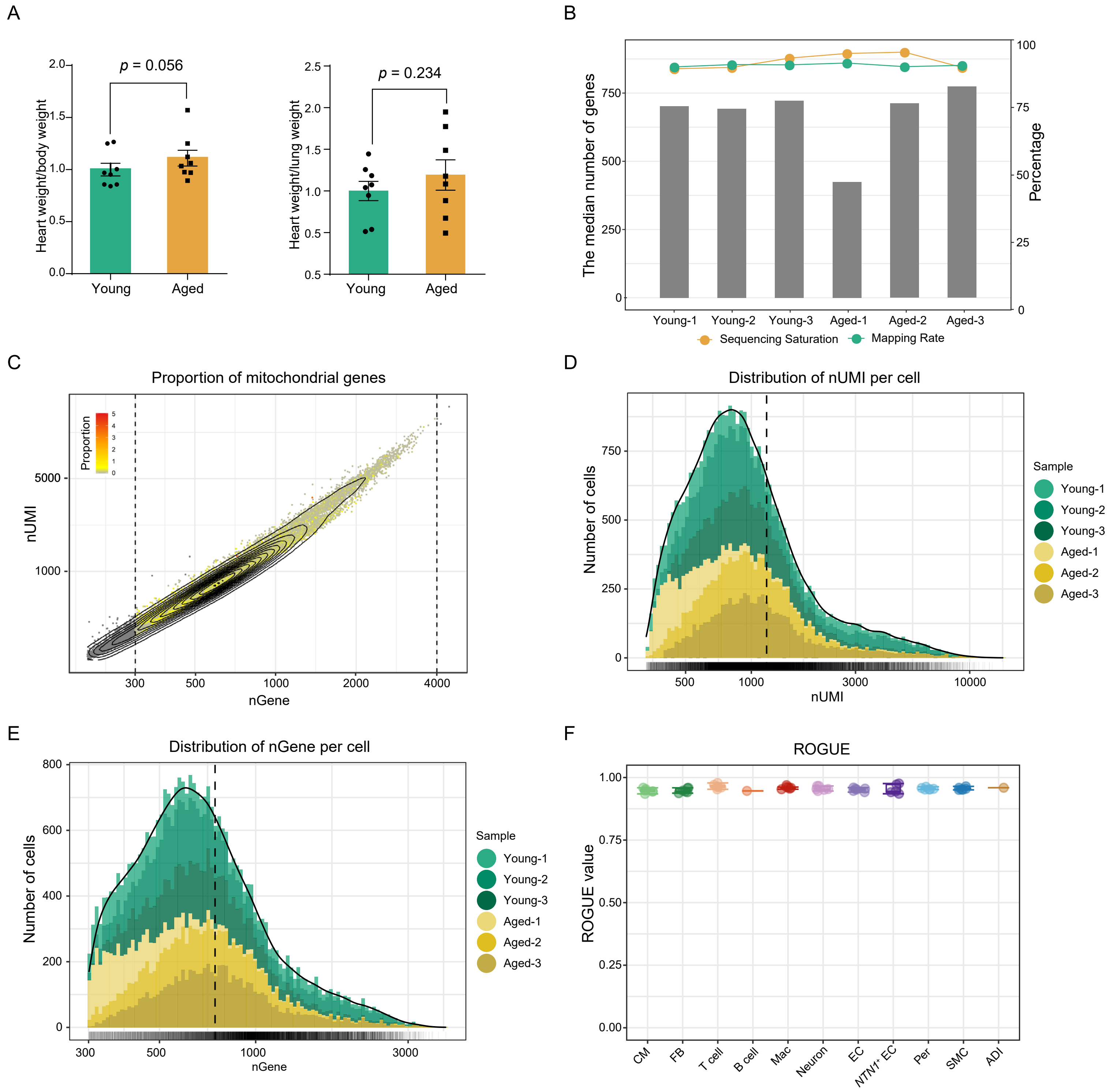
A
p = 0.056
Heart weight/body weight
Young
Aged
p = 0.234
Heart weight/lung weight
Young
Aged
B
The median number of genes
Percentage
Young-1
Young-2
Young-3
Aged-1
Aged-2
Aged-3
Sequencing Saturation
Mapping Rate
C
Proportion of mitochondrial genes
Proportion
nUMI
nGene
D
Distribution of nUMI per cell
Number of cells
nUMI
Sample
Young-1
Young-2
Young-3
Aged-1
Aged-2
Aged-3
E
Distribution of nGene per cell
Number of cells
nGene
Sample
Young-1
Young-2
Young-3
Aged-1
Aged-2
Aged-3
F
ROGUE
ROGUE value
CM
FB
T cell
B cell
Mac
Neuron
EC
NTN1+ EC
Per
SMC
ADI

Figure S2

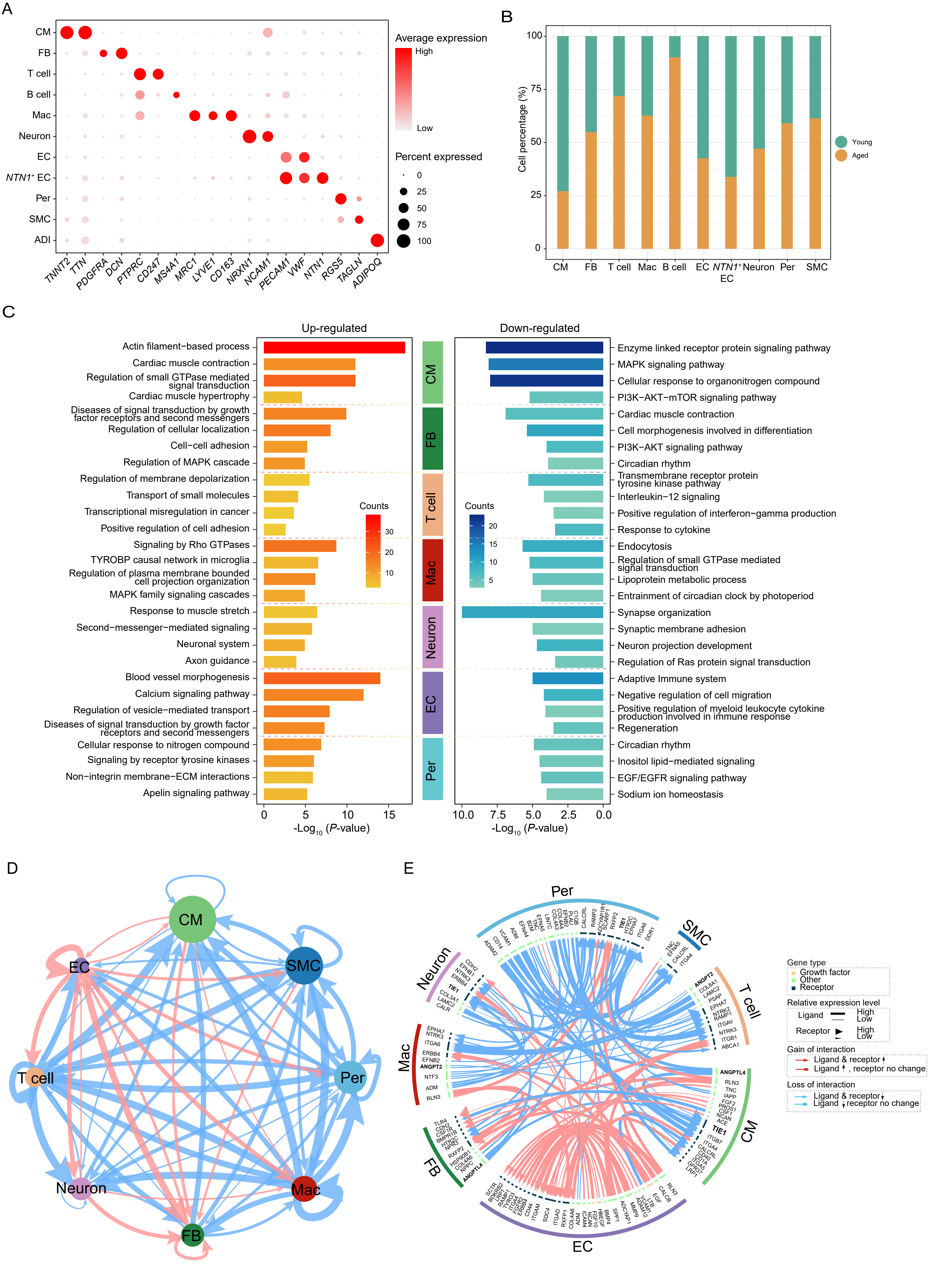
A
CM
FB
T cell
B cell
Mac
Neuron
EC
NTN1⁺ EC
Per
SMC
ADI
TNNT2
TTN
PDGFRA
DCN
PTPRC
CD247
MS4A1
MRC1
LYVE1
CD163
NRXN1
NCAM1
PECAM1
VWF
NTN1
RGS5
TAGLN
ADIPOQ
Average expression
High
Low
Percent expressed
0
25
50
75
100
B
Cell percentage (%)
0
25
50
75
100
CM
FB
T cell
Mac
B cell
EC
NTN1⁺ EC
Neuron
Per
SMC
Young
Aged
C
Up-regulated
Down-regulated
Actin filament−based process
Cardiac muscle contraction
Regulation of small GTPase mediated signal transduction
Cardiac muscle hypertrophy
Diseases of signal transduction by growth factor receptors and second messengers
Regulation of cellular localization
Cell−cell adhesion
Regulation of MAPK cascade
Regulation of membrane depolarization
Transport of small molecules
Transcriptional misregulation in cancer
Positive regulation of cell adhesion
Signaling by Rho GTPases
TYROBP causal network in microglia
Regulation of plasma membrane bounded cell projection organization
MAPK family signaling cascades
Response to muscle stretch
Second−messenger−mediated signaling
Neuronal system
Axon guidance
Blood vessel morphogenesis
Calcium signaling pathway
Regulation of vesicle−mediated transport
Diseases of signal transduction by growth factor receptors and second messengers
Cellular response to nitrogen compound
Signaling by receptor tyrosine kinases
Non−integrin membrane−ECM interactions
Apelin signaling pathway
Counts
30
20
10
CM
FB
T cell
Mac
Neuron
EC
Per
Counts
20
15
10
5
Enzyme linked receptor protein signaling pathway
MAPK signaling pathway
Cellular response to organonitrogen compound
PI3K−AKT−mTOR signaling pathway
Cardiac muscle contraction
Cell morphogenesis involved in differentiation
PI3K−AKT signaling pathway
Circadian rhythm
Transmembrane receptor protein tyrosine kinase pathway
Interleukin−12 signaling
Positive regulation of interferon−gamma production
Response to cytokine
Endocytosis
Regulation of small GTPase mediated signal transduction
Lipoprotein metabolic process
Entrainment of circadian clock by photoperiod
Synapse organization
Synaptic membrane adhesion
Neuron projection development
Regulation of Ras protein signal transduction
Adaptive Immune system
Negative regulation of cell migration
Positive regulation of myeloid leukocyte cytokine production involved in immune response
Regeneration
Circadian rhythm
Inositol lipid−mediated signaling
EGF/EGFR signaling pathway
Sodium ion homeostasis
0
5
10
15
-Log10 (P-value)
10.0
7.5
5.0
2.5
0.0
-Log10 (P-value)
D
CM
EC
SMC
T cell
Per
Neuron
Mac
FB
E
Per
SMC
T cell
CM
EC
FB
Mac
Neuron
Gene type
Growth factor
Other
Receptor
Relative expression level
Ligand
High
Low
Receptor
High
Low
Gain of interaction
Ligand & receptor ↑
Ligand ↑, receptor no change
Loss of interaction
Ligand & receptor ↓
Ligand ↓ receptor no change

Figure S3

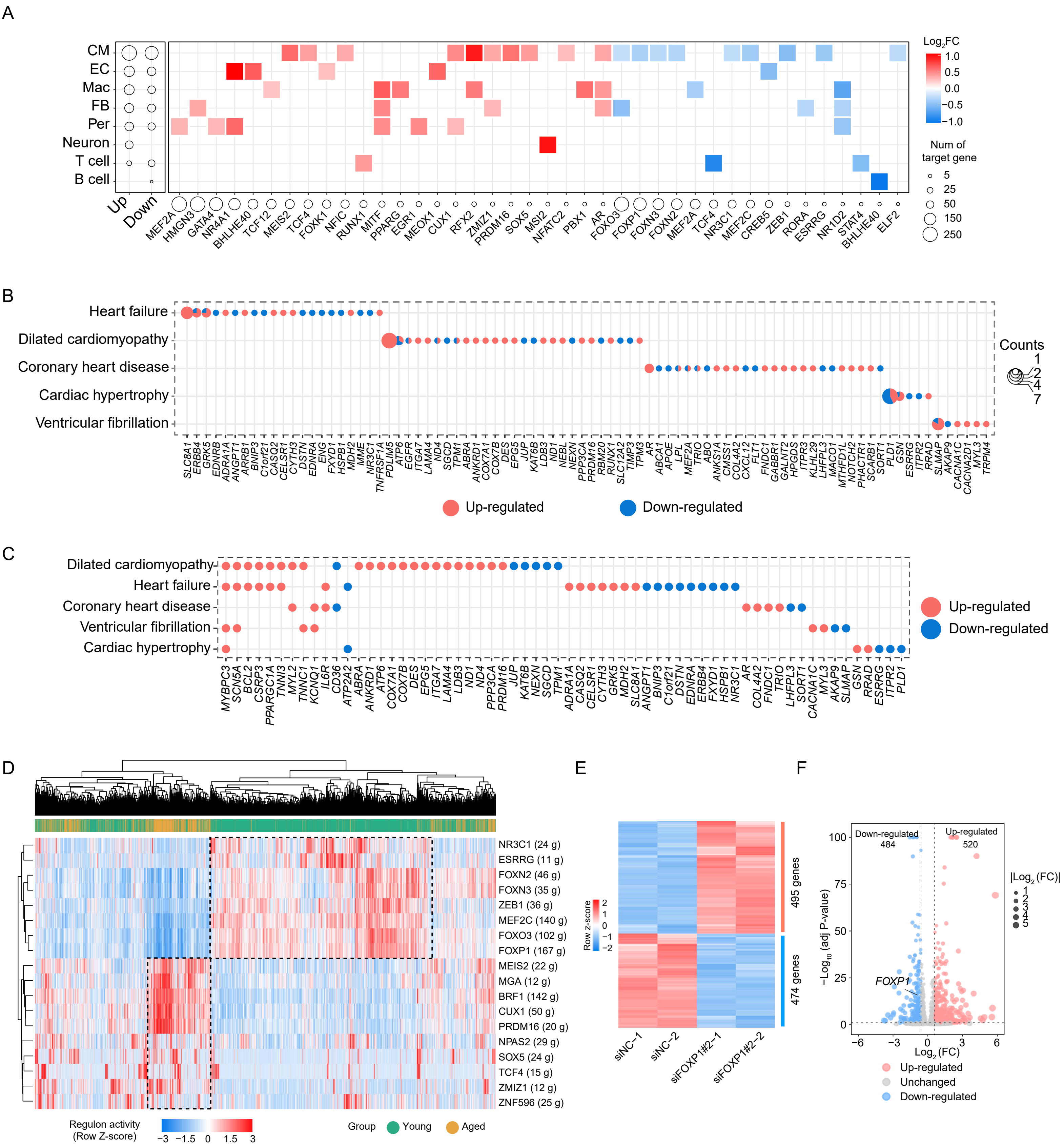

A
CM
EC
Mac
FB
Per
Neuron
T cell
B cell
Up
Down
MEF2A HMGN3 GATA4 NR4A1 BHLHE40 TCF12 MEIS2 TCF4 FOXK1 NFIC RUNX1 MITF PPARG EGR1 MEOX1 CUX1 RFX2 ZMIZ1 PRDM16 SOX5 MSI2 NFATC2 PBX1 AR FOXO3 FOXP1 FOXN3 FOXN2 MEF2A TCF4 NR3C1 MEF2C CREB5 ZEB1 RORA ESRRG NR1D2 STAT4 BHLHE40 ELF2
Log2FC
1.0
0.5
0.0
−0.5
−1.0
Num of target gene
5
25
50
150
250
B
Heart failure
Dilated cardiomyopathy
Coronary heart disease
Cardiac hypertrophy
Ventricular fibrillation
Counts
1
2
4
7
Up-regulated
Down-regulated
C
Dilated cardiomyopathy
Heart failure
Coronary heart disease
Ventricular fibrillation
Cardiac hypertrophy
Up-regulated
Down-regulated
D
NR3C1 (24 g)
ESRRG (11 g)
FOXN2 (46 g)
FOXN3 (35 g)
ZEB1 (36 g)
MEF2C (140 g)
FOXO3 (102 g)
FOXP1 (167 g)
MEIS2 (22 g)
MGA (12 g)
BRF1 (142 g)
CUX1 (50 g)
PRDM16 (20 g)
NPAS2 (29 g)
SOX5 (24 g)
TCF4 (15 g)
ZMIZ1 (12 g)
ZNF596 (25 g)
Regulon activity (Row Z-score)
−3 −1.5 0 1.5 3
Group Young Aged
E
Row z-score
495 genes
474 genes
siNC−1
siNC−2
siFOXP1#2−1
siFOXP1#2−2
F
Down-regulated 484
Up-regulated 520
FOXP1
−Log10 (adj P-value)
Log2 (FC)
|Log2 (FC)|
Up-regulated
Unchanged
Down-regulated
